# ﻿Updating the diversity: three novel species of *Triblidium* (Triblidiaceae, Rhytismatales) in west Yunnan, China

**DOI:** 10.3897/mycokeys.121.165642

**Published:** 2025-09-01

**Authors:** Cui-Jin-Yi Li, Qi Zhao, Prapassorn Damrongkool Eungwanichayapant, Feng-Ming Yu, Kevin David Hyde, Kandawatte Wedaralalage Thilini Chethana, Wei-Wei Liu, Dong-Mei Liu

**Affiliations:** 1 Institue of Ecology, Chinese Research Academy of Environmental Sciences, Beijing 100012, China Chinese Research Academy of Environmental Sciences Beijing China; 2 State Key Laboratory of Phytochemistry and Natural Medicines, Kunming Institute of Botany, Chinese Academy of Sciences, Kunming, Yunnan 650201, China Mae Fah Luang University Chiang Rai Thailand; 3 Center of Excellence in Fungal Research, Mae Fah Luang University, Chiang Rai, 57100, Thailand Chinese Academy of Sciences Yunnan China; 4 School of Science, Mae Fah Luang University, Chiang Rai, 57100, Thailand Chinese Academy of Sciences Kunming China

**Keywords:** Phylogeny, Rhytismatales, three new taxa, taxonomy, Triblidiaceae

## Abstract

During a survey of discomycetes in Yunnan, China, three saprobic species of *Triblidium* were discovered on decayed wood and the bark of living oak trees. These species are characterised by black cleistohymenial apothecia with 6–8 teeth-like lobes, which can be either stipitate or sessile, with greyish-white to orange hymenium, a well-developed covering and basal stroma, consisting of carbonised to hyaline angular cells or hyaline hyphae, with a subhymenium composed of hyaline angular cells, clavate, J- asci, with an elliptical or rounded apex and long acicular and ellipsoidal ascospores with multiple septa. *Triblidium longisporum*, *T. stipitatum* and *T. daliense* are described as new species within *Triblidium*, supported by both morphological features and phylogenetic analyses of the LSU-ITS-mtSSU dataset. Detailed descriptions, illustrations and multi-gene analyses fully support each species.

## ﻿Introduction

Triblidiaceae Rehm was established within the suborder Triblidieae [Tryblidieae] under Pezizaceae (Discomycetes) by Rehm (1887–1896). It initially included two genera: *Triblidium* Rebent. and *Tryblidiopsis* P. Karst. (Rehm 1887–1896). Rehm (1912) included *Pseudographis* Nyl. and *Tryblidiella* Sacc in this family. Von Höhnel (1918) further broadened the concept of Triblidiaceae, encompassing 11 genera. Nannfeldt (1932) identified *Pseudographis* as a transitional taxon between Discomycetes and apothecial lichens. Based on its thick-walled epithecium and asci, *Pseudographis* was re-assigned to Lecanorales (Nannfeldt 1932). Subsequently, Triblidiaceae was placed within Ostropales Nannf. (Lecanoromycetes) due to the characteristic reddish-purple, iodine reaction observed in *Pseudographis* ascospores (Hawksworth et al. 1983; Sherwood-Pike 1987). Over time, this reclassification led to only *Pseudographis* and *Triblidium* retained within the family, while *Tryblidiopsis* was transferred to Rhytismatales (Hawksworth et al. 1983; Sherwood-Pike 1987). Eriksson (1992) introduced the genus *Huangshania* O.E. Erikss. and placed it within Triblidiaceae under a separate order, Triblidiales, to reflect the distant relationship between *Triblidium* and *Graphis* Adans., the type genus of Graphidales. Magnes (1997) extensively revised the family, re-distributing certain species into 22 genera across seven families. Additionally, he proposed synonymising Triblidiales with Rhytismatales, based on similarities in ascomatal development and ascus structures, rejecting any significant relationship between Triblidiaceae and Graphidaceae (Magnes 1997).

Phylogenetic analyses later confirmed the significant divergence of *Pseudographis* from *Triblidium* and *Huangshania* (Karakehian et al. 2019). As a result, only *Triblidium* and *Huangshania* were retained, while Triblidiales was synonymised under Rhytismatales (Karakehian et al. 2019). Based on morphological similarities, distinguished primarily by ascospore differences and to preserve the monophyly of *Triblidium*, *Huangshania* was synonymised under *Triblidium* following a four-locus combined (ITS-LSU-mtSSU-*rpb*2) phylogenetic analysis. However, only its type species, *Huangshania verrucosa* O.E. Erikss., was formally transferred, as molecular data for the remaining species were unavailable (Lv et al. 2019). Currently, Triblidiaceae comprises only the type genus, *Triblidium* and belongs in Triblidiaceae, Rhytismatales, Leotiomycetes (Hyde et al. 2024).

*Triblidium* was first established by Rebentisch in 1805 with *T. caliciiforme* Rebent. as the type. Later, Rehm (1887–1896, 1912) designated it as the type genus of the family Triblidiaceae. *Triblidium* is saprobic and occurs on the bark of Pinaceae, Ericaceae and Fagaceae, with Magnes (1997) suggesting the possibility of an endophytic phase. The genus is characterised by circular to rectangular, erumpent apothecia with a rough surface and irregular splits, carbonised cells or hyphae in the covering stroma, intricate hyphae or angular cells in the basal stroma, hyaline hyphae within the internal matrix of stroma, intricate hyphae or angular cells at the subhymenium, filiform paraphyses, covered with a thin gelatinous sheath and lack of swelling at the apex, sequentially ripening asci with J- apex and elliptical, muriform and phragmosporous ascospores that lack a gelatinous sheath (Eriksson 1992; Magnes 1997; Karakehian et al. 2019; Lv et al. 2019; Guo et al. 2024). A total of 11 species have been described across America, Asia and Europe (Eriksson 1992; Magnes 1997; Karakehian et al. 2019; Lv et al. 2019; Guo et al. 2024), with seven species recorded in China (Eriksson 1992; Karakehian et al. 2019; Lv et al. 2019; Guo et al. 2024).

During a survey of Leotiomycetes diversity in western Yunnan Province, China (Ekanayaka et al. 2019; Li et al. 2022a, b, 2024a, b; Luo et al. 2024, 2025a, b; Su et al. 2022, 2023, 2025), five specimens were collected from decayed branches and the bark of living oak trees. These specimens were identified as three novel species of *Triblidium*. Detailed morphological descriptions, accompanying illustrations and phylogenetic analyses of the new species are presented in this study.

## ﻿Materials and methods

### ﻿Sample collection and morphological studies

During the field investigations from 2021 to 2024, all specimens were obtained from Dali City, Yunnan Province, China. Our collections were obtained from protected areas and primary forests at altitudes ranging from 2,380 m to 2,600 m. Sampling methods followed those described by Li et al. (2024b). The dried specimens are deposited at the Herbarium of Cryptogams, Kunming Institute of Botany, Academia Sinica (HKAS). Facesoffungi numbers were obtained, based on Jayasiri et al. (2015), while Index Fungorum numbers were obtained following the guidelines provided by Index Fungorum (2025). Data on the taxa were also deposited in the Greater Mekong Subregion database (Chaiwan et al. 2021).

Fresh apothecia were photographed in the field with a Canon EOS M100 camera (Canon Co. LTD, Japan). Macro-morphological features of dried apothecia were captured using a Canon EOS 70D(W) digital camera mounted on a C-PSN stereomicroscope (Nikon Corporation, Tokyo, Japan). Both fresh and dried apothecia were manually sliced with razor blades for observation under a charge-coupled device (CCD) SC 2000C attached to a Nikon ECLIPSE Ni-U compound microscope (Nikon Corporation, Tokyo, Japan). Vertical sections were examined to study the excipulum and hymenium, while mature apothecia, squashed in water and 2% potassium hydroxide (KOH) solution, were used to observe asci, ascospores and paraphyses. The blue iodine reaction at the ascus apex was tested with Melzer’s reagent, both in water and 2% KOH solution. All measurements were made using Tarosoft® Image Framework (IFW) and adjusted in Adobe Photoshop 2020 (Adobe Systems, USA). The Q value represents the ratio of ascospore length to width, with ‘n’ indicating the number of measured structures and Qm denoting the mean Q value. Colour references for the apothecia, hymenium and excipulum were sourced from https://www.colorhexa.com/.

### ﻿DNA extraction, PCR amplification and sequencing

Genomic DNA was extracted from multiple mature fruiting bodies using the Trilief™ Plant Genomic DNA Kit (Tsingke Biological Technology Co., Ltd, Beijing, China) following cleaning with sterilised water and a 75% alcohol solution. The amplification primers for each gene were as follows: ITS1-F and ITS4 for the nuclear internal transcribed spacers (ITS) (White et al. 1990; Gardes and Bruns 1993); LR0R and LR5 for the D1/D2 domain of the nuclear large subunit ribosomal RNA (LSU) (Vilgalys and Hester 1990); mrSSU1 and mrSSU3R for the mitochondrial small subunit (mtSSU rDNA) (Zoller et al. 1999). The 25 μl total reaction volume consisted of 12.5 μl of 2× Power Taq PCR MasterMix, 7.5 μl of sterile deionised water, 1 μl of each primer (100 μM stock) and 3 μl of DNA template. PCR amplification was performed using a TC-type gene amplifier (LifeECO, Hangzhou Bori Technology Co., LTD, Hangzhou, China) with the following conditions: initial denaturation at 94 °C for 5 min, followed by 35 cycles of denaturation at 94 °C for 20 s, annealing at 53 °C for 30 s (for LSU and ITS) or 56 °C for mtSSU, extension at 72 °C for 45 s and final extension at 72 °C for 10 min. PCR products were verified by electrophoresis on a 1% agarose gel, stained with TS-GelRed Ver. 2 (Tsingke Biological Technology Co., Ltd, Beijing, China) and subsequently sequenced at Tsingke Biological Technology Co., Ltd.

### ﻿Sequence assembly and alignment

The forward and reverse sequences were assembled using ContigExpress (Invitrogen, USA) and edited in BioEdit 7.2.5.0 (Hall 1999). The newly-generated sequences were deposited in GenBank (Table 1) and homologous sequences were identified through a BLASTn search against the GenBank database. Phylogenetic analyses included related sequences from GenBank, with *Fanglania hubeiensis* (Hou 1406) and *F. parasiticum* (HOU 1417) as the outgroup taxa (Guo et al. 2024). Sequence alignment for LSU, ITS and mtSSU was performed using the MAFFT 7 online server (https://mafft.cbrc.jp/alignment/server/) (Katoh et al. 2019) with default settings and manually refined in BioEdit. The datasets were trimmed using TrimAl v.1.3, applying the “gt 0.5” option for LSU and ITS and the ‘gappyout’ option for mtSSU (Capella-Gutiérrez et al. 2009). Final datasets for each gene were concatenated into a single combined dataset in the order ‘LSU-ITS-mtSSU’, using SequenceMatrix 1.7.8 (Vaidya et al. 2011) and the file format was converted from “.fasta” to “.nexus” using the Alignment Transformation Environment online tool (https://www.sing-group.org/ALTER/). Herbarium abbreviations used in Table 1: CNUCC (Capital Normal University Culture Collection Center), FH (Harvard University), GJO (Universalmuseum Joanneum), HKAS (Herbarium of Cryptogams, Kunming Institute of Botany), PRA (Czech Academy of Sciences), UCH (Universidad Autónoma de Chiriquí) and UME (Umeå University).

**Table 1.** Detailed information and corresponding GenBank accession numbers for the taxa utilised in the phylogenetic analyses of this study are provided. ‘^†^’ denotes type species, ‘*’ denotes holotypes, newly-generated sequences in bold font and ‘-’ indicates that sequence data are unavailable.

Taxon name	Voucher	Gene accession No.			Reference
		ITS	LSU	mtSSU	
*Fanglania hubeiensis* ^†*^	HOU 1406	OQ944273	OQ944311	–	Guo et al. (2024)
Fanglania parasitica*	HOU 1417	OQ944274	OQ944312	OQ944353	Guo et al. (2024)
*Neorhytisma panamense*	HOU 601	GQ253102	GQ253099	OQ944337	Hou et al. (2010)
*Neorhytisma panamense* ^†^	UCH 5284	OQ944277	–	OQ944356	Wang et al. (2023)
*Pseudographis elatina*	GJO-0090016	MK751794	MK751803	MK751717	Karakehian et al. (2019)
*Pseudographis elatina*	PRA-Vondrak25131	OQ718032	–	OQ646404	Vondrák et al. (2023)
*Pseudographis pinicola*	FH-18061706	MK751795	MK751804	MK751718	Karakehian et al. (2019)
*Pseudographis pinicola*	FH:NB842	MK751796	MK751805	MK751719	Karakehian et al. (2019)
Shuqunia clavata*	HOU 1812	PP488619	–	–	Guo et al. (2024)
*Shuqunia longa*	HOU 457A	PP488621	PP488722	PP488816	Guo et al. (2024)
*Shuqunia longa* ^†*^	HOU 368B	PP488620	PP488721	PP488815	Guo et al. (2024)
*Shuqunia nitens*	HOU 1845	PP488624	PP488725	PP488819	Guo et al. (2024)
*Shuqunia nitens*	HOU 1758Y	PP488623	PP488724	PP488818	Guo et al. (2024)
*Shuqunia nitens*	CNUCC 1758	–	PP488666	PP488761	Guo et al. (2024)
Shuqunia nitens*	HOU 1758	PP488622	PP488723	PP488817	Guo et al. (2024)
*Shuqunia rhododendri*	CNUCC 1848C.1	PP488554	PP488667	PP488762	Guo et al. (2024)
*Shuqunia rhododendri*	CNUCC 1848C.2	PP488625	PP488726	PP488820	Guo et al. (2024)
Shuqunia rhododendri*	HOU 1848D	PP488626	PP488727	PP488821	Guo et al. (2024)
*Shuqunia yunnanensis*	HOU 1566	PP488627	PP488728	PP488822	Guo et al. (2024)
*Shuqunia yunnanensis*	HOU 1572	PP488629	PP488730	PP488824	Guo et al. (2024)
*Shuqunia yunnanensis*	HOU 1573	PP488630	–	PP488825	Guo et al. (2024)
Shuqunia yunnanensis*	HOU 1567	PP488628	PP488729	PP488823	Guo et al. (2024)
*Triblidium caliciiforme* ^†^	-	MK751798	MK751807	MK751721	Karakehian et al. (2019)
*Triblidium daliense* ^†*^	**HKAS 128302**	**PV590251**	**PV594532**	**PV593644**	**This study**
***Triblidium daliense***	**HKAS 145635**	**PV590289**	**PV594530**	**PV593645**	**This study**
Triblidium hubeiense*	HOU 1350A	MN541813	MN541811	MN541828	Lv et al. (2019)
*Triblidium laojunshanense*	HOU 2123	PP508362	PP505428	PP505443	Guo et al. (2024)
Triblidium laojunshanense*	HOU 1620	PP488635	PP488734	PP488830	Guo et al. (2024)
*Triblidium longisporum* ^†*^	**HKAS 145642**	**PV602119**	**PV594533**	**PV593649**	**This study**
***Triblidium longisporum***	**HKAS 145640**	**PV602120**	**PV594528**	**PV593648**	**This study**
*Triblidium rhododendri*	HOU 1848B	PP488636	PP488735	PP488831	Guo et al. (2024)
Triblidium rhododendri*	HOU 326A	PP488637	PP488736	PP488832	Guo et al. (2024)
*Triblidium rostriforme*	1	–	MN541820	MN541821	Lv et al. (2019)
*Triblidium sichuanense*	HOU 1964	PP508363	PP505429	PP505444	Guo et al. (2024)
Triblidium sichuanense*	HOU 295	PP488639	PP488738	PP488834	Guo et al. (2024)
***Triblidium* sp.**	**HKAS 128327**	**PV594526**	**PV594527**	–	**This study**
*Triblidium stipitatum* ^†*^	**HKAS 145641**	**PV594524**	**PV594531**	**PV593647**	**This study**
***Triblidium stipitatum***	**HKAS 145639**	**PV594525**	**PV594529**	**PV593646**	**This study**
*Triblidium verrucosum*	UME-29336a	MK751793	MK751802	MK751716	Karakehian et al. (2019)
*Triblidium yunnanense*	HOU 1611	PP488640	PP488739	PP488835	Guo et al. (2024)
*Triblidium yunnanense*	HOU 1179	MN541814	MN541809	MN541816	Lv et al. (2019)
*Tryblidiopsis pinastri* ^†^	Lantz 412	–	HM140573	–	Lantz et al. (2011)
*Tryblidiopsis sichuanensis*	HOU 300	KC312677	KC312679	KC312693	Wang et al. (2014)
Tryblidiopsis sichuanensis*	HOU 306	KC312676	KC312683	KC312692	Wang et al. (2014)
Tryblidiopsis sinensis*	HOU 814	KC312674	KC312681	KC312694	Wang et al. (2014)

### ﻿Phylogenetic analyses

Maximum Likelihood analysis was performed using the IQ-Tree web portal (http://iqtree.cibiv.univie.ac.at/). The optimal substitution models for each gene were automatically determined, based on the provided partition file. Clade support was assessed using a 1,000-replicate SH-aLRT test and the ultrafast bootstrap (UFB) method (Guindon et al. 2010; Hoang et al. 2018). Bayesian Inference (BI) was conducted using MrBayes v.3.1.2. Posterior probabilities (PP) were estimated via Markov Chain Monte Carlo (MCMC) sampling.

The general time-reversible model with a discrete gamma distribution, coupled with a proportion of an invariant (GTR+I+G) was selected for LSU and mtSSU and the unequal transition rates and unequal base frequency model with a discrete gamma distribution coupled with a proportion of an invariant (HKY+I+G) was selected for ITS using MrModelTest 2.3 (Nylander et al. 2008). Four simultaneous chains were run for 2,000,000 generations, with tree sampling at every 100^th^ generation. An average standard deviation of split frequencies below 0.01 indicated convergence. The first 25% of trees were discarded as burn-in and the remaining trees were used to estimate PP in the majority-rule consensus tree. A PP ≥ 0.90 indicated strong support. The phylogenetic tree was visualised using FigTree 1.4.0 (Rambaut 2009), illustrated with Adobe Illustrator 2020 and Photoshop 2020 (https://www.adobe.com/). The combined alignment was deposited at TreeBASE (submission ID: 32283).

## ﻿Results

### ﻿Phylogenetic analyses

**Figure 1.**
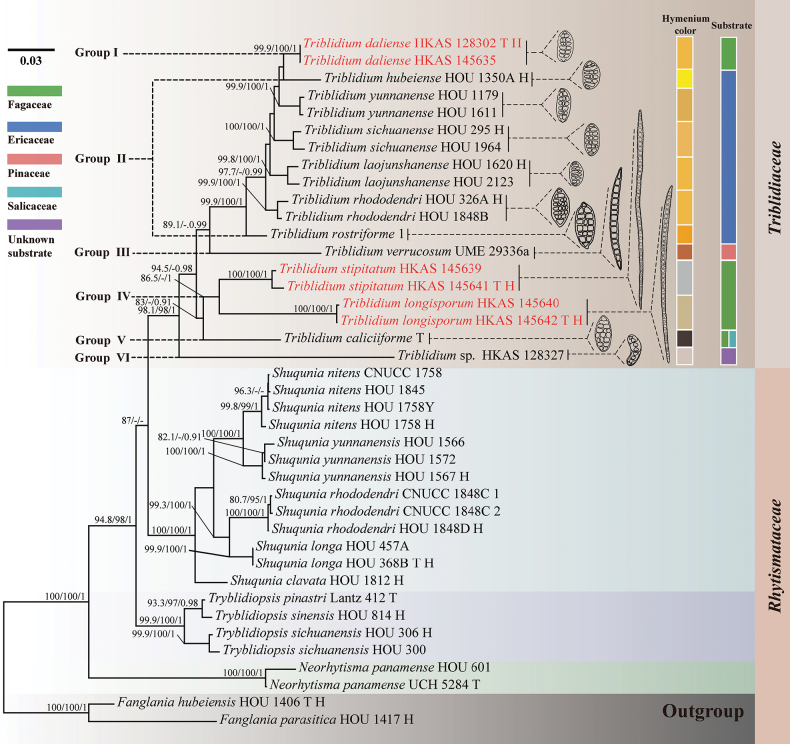
Maximum Likelihood tree, based on the LSU, ITS and mtSSU sequences, showing the phylogenetic position of *Triblidium*. Bootstrap support values for ML ≥ 80 of SH-aLRT or ML > 95 of UFB and posterior probability for BI ≥ 0.90 are indicated above the nodes and separated by “-/-/-” (SH-aLRT/UFB/PP). The newly-generated isolates of the current study are highlighted in red, whereas types species within the genera are denoted with ‘T’ and holotypes are denoted as ‘H’ following the strain number. Hymenium colours refer to the illustrations from Lv et al. (2019), Karakehian et al. (2019), Guo et al. (2024) and this study.

The combined LSU, ITS and mtSSU dataset consists of 23 taxa, including 40 isolates and 2169 aligned nucleotide sites, with the LSU region comprising 856 bp, the ITS region comprising 533 bp and the mtSSU region comprising 780 bp with gaps. The combined alignment comprised 552 parsimony-informative characters, 179 singleton sites and 1438 constant characters. The ML and BI analyses yielded similar topologies. The Maximum Likelihood matrix had 710 distinct alignment patterns with 8.48% undetermined characters or gaps. The best Maximum Likelihood tree, with a final likelihood value of -11567.119, is shown in Fig. 1. The topology of the phylogenetic tree, based on the LSU-ITS-mtSSU dataset in this study, closely resembles the family tree presented by Guo et al. (2024).

*Triblidium longisporum* and *T. stipitatum* formed an individual clade within *Triblidium* close to *T. caliciiforme* with the Maximum Likelihood bootstrap support of 83% in the SH-aLRT test, 81% in the UFB method and the Bayesian posterior probability of 0.91 (Fig. 1). *Triblidium daliense* is closely related to *T. hubeiense*, with the Maximum Likelihood bootstrap support of 79.9% in the SH-aLRT test, 80% in the UFB method and the Bayesian posterior probability of 0.90 (Fig. 1). The guidelines of Chethana et al. (2021) and Guo et al. (2024) were followed in determining whether we had new taxa or records.

### ﻿Taxonomy

#### ﻿ Triblidium daliense

Taxon classificationFungiRhytismatalesTriblidiaceae

C.J.Y. Li, K.W.T. Chethana & Q. Zhao sp. nov.

0FC83F86-D398-52C0-8A01-8AD19564B9B3

Index Fungorum: IF904194

Facesoffungi Number: FoF18050

Fig. 2

##### Etymology.

The specific epithet refers to Dali City, where the type specimen was collected.

**Figure 2.**
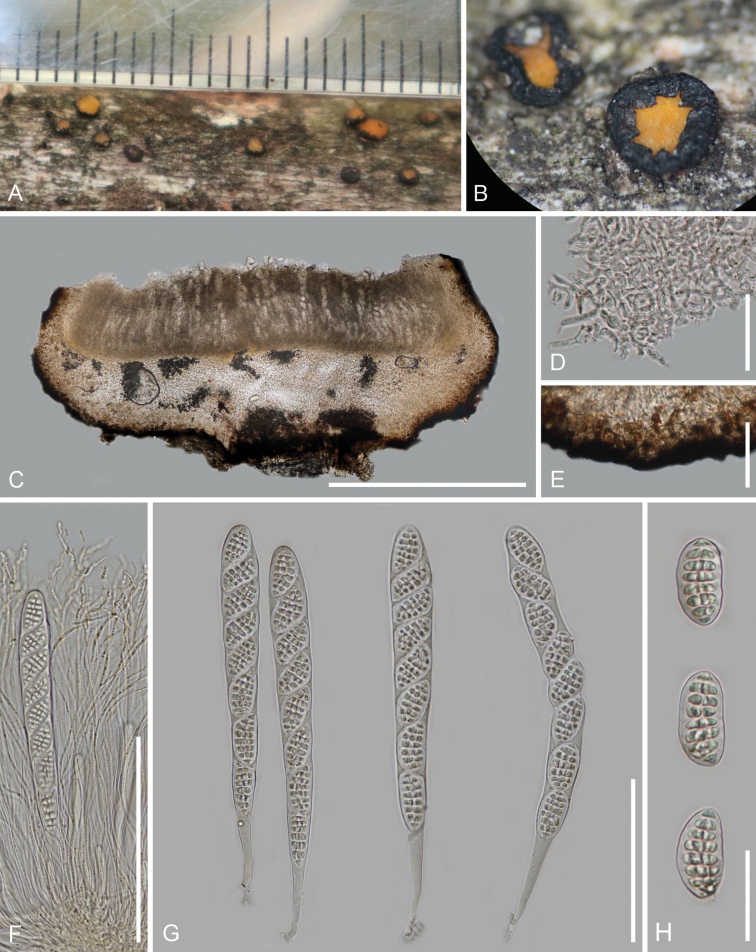
*Triblidium daliense* (HKAS 128302, holotype). A. Fresh apothecia on the substrate; B. Dried apothecia on the substrate; C. Vertical section of an apothecium; D. Medullary excipulum; E. Ectal excipulum; F. Paraphyses; G. Asci; H. Ascospores. Scale bars: 500 μm (C); 35 μm (D, E); 150 μm (F); 100 μm (G); 30 μm (H).

##### Holotype.

HKAS 128302.

##### Diagnosis.

It is similar to *T. sichuanense*, but *T. daliense* has larger asci, wider paraphyses, thicker internal matrix of stroma and the inner layers of the covering and basal stroma consisting of hyaline hyphae.

##### Description.

Saprobic on the bark of the fallen branches of Fagaceae. **Sexual morph**: ***Apothecia*** 0.9–1.6 mm wide (*x̄* = 1.2 mm, n = 20) when fresh, 1.1–1.6 mm wide (*x̄* = 1.2 mm, n = 20), 0.3–0.5 mm high (*x̄* = 0.4 mm, n = 10) when dried, scattered, superficial, discoid, sessile, erumpent from the bark, initially growing as a cleistohymenial development, the hymenium tightly protected by excipulum when immature, splitting to expose hymenium by usually irregular 6–8 teeth-like lobes in the surface in a humid environment, black (#4a4750) surface with polygonal areolae. ***Discs*** flat to slightly raised, circular, orange (#c4892d) when fresh, bright orange (#db9938) when dried. ***Receptacles*** rough and black when fresh, sculptured with polygonal areolae or wrinkled on the surface when dried. ***Covering stroma*** 54–138 μm thick, comprised of carbonised textura angularis cells and the inner layers of hyaline hyphae. ***Hymenium*** 200–236 μm (*x̄* = 216 μm, n = 10) thick, hyaline to pale brown. ***Subhymenium*** 30–50 μm (*x̄* = 39 μm, n = 15) thick, comprised of hyaline, textura angularis cells, 3.3–7.9 μm (*x̄* = 5.3 μm, n = 40) in diam. ***Internal matrix of stroma*** 55–215 μm (*x̄* = 123 μm, n = 40) thick, well-developed, comprised of hyaline, textura intricata hyphae, 1.6–2.9 μm (*x̄* = 2.2 μm, n = 40) in diam., non-gelatinous. ***Basal stroma*** 60–95 μm (*x̄* = 74 μm, n = 40) thick, well-developed, the outer layers comprised of carbonised, very dark brown to black, textura angularis cells, 4.3–10.1 μm (*x̄* = 6.7 μm, n = 40) in diam., the inner layers comprised of highly melanised hyaline hyphae, 2.9–5.5 μm (*x̄* = 3.8 μm, n = 40) in diam. ***Paraphyses*** 270–330 × 2.1–3.3 μm (*x̄* = 290 × 2.7 μm, n = 50) wide, hyaline with golden oil drops, filiform, apically irregular-curved and occasionally branched, aseptate. ***Asci*** ripening sequentially, 235–292 × 23–28 μm (*x̄* = 257 × 24 μm, n = 25), unitunicate, 8-spored, clavate, apically rounded without an amyloid reaction in Melzer’s reagent, tapering to a fragile pleurorhynchous subtruncate base, croziers absent. ***Ascospores*** 23.4–35.5 × 11.8–16.5 μm (*x̄* = 26.7 × 13.4 μm, n = 55, Q = 1.6–2.2, Qm = 2.0 ± 0.1), overlapping uniseriate, ellipsoidal and muriform, hyaline, smooth, slightly curved, eight transverse septa and one or two longitudinal and oblique septa, without a gelatinous sheath. **Asexual morph**: Undetermined.

##### Material examined.

China • Yunnan Province, Dali City, Eryuan County, Ma’an Mountain, altitude 2,600 m, on the bark of the fallen branches of Fagaceae, 26 July 2021, Cuijinyi Li, LCJY-209 (HKAS 128302, holotype); • ibid., Cuijinyi Li, LCJY-209-2 (HKAS 145635, isotype).

##### Notes.

Our collection was placed sister to *T. hubeiense*, with the Maximum Likelihood bootstrap support of 79.9% in the SH-aLRT test, 80% in the UFB method and a Bayesian posterior probability of 0.9 (Fig. 1). *Triblidium daliense* can be distinguished from *T. hubeiense* by its smaller apothecia, thinner covering stroma (54–138 μm vs. 270–300 μm), thinner basal stroma (60–95 μm vs. 65–160 μm), carbonised angular cells at the outer layer, wider paraphyses (2.1–3.3 μm vs. ca. 1 μm) with branched tips and larger asci (235–292 × 23–28 μm vs. 160–200 × 15–24 μm) in contrast to the melanised hyphae of the latter species at the outer layers (Lv et al. 2019). The most morphologically similar species to our species is *T. sichuanense*, which is distinguished by the presence of angular cells in the inner layers of the covering stroma and basal stroma, a thinner internal matrix of the stroma (50–80 μm vs. 55–215 μm), thinner paraphyses (1 μm vs. 2.1–3.3 μm) and smaller asci (120–220 × 12–20 μm vs. 235–292 × 23–28 μm) (Guo et al. 2024). Based on the molecular analyses, the ITS sequence of *T. daliense* exhibited a 3.3% difference with no gaps (16/485) to *T. yunnanense* (isolate: HOU1822B), while the LSU sequence showed a 2.9% difference with six gaps (26/898) to *T. yunnanense* (isolate: HOU875A) and the mtSSU sequence displayed a 0.72% difference with no gaps (6/830) to *T. hubeiense* (isolate: HOU1350A).

#### ﻿ Triblidium longisporum

Taxon classificationFungiRhytismatalesTriblidiaceae

C.J.Y. Li, K.W.T. Chethana & Q. Zhao sp. nov.

42ECA05E-184D-5D39-A964-9FADE16B261D

Index Fungorum: IF904195

Facesoffungi Number: FoF18051

Fig. 3

##### Etymology.

The specific epithet refers to the long ascospores, ‘*longi*’ (lat.) = long, ‘*sporum*’ (lat.) = spore.

**Figure 3.**
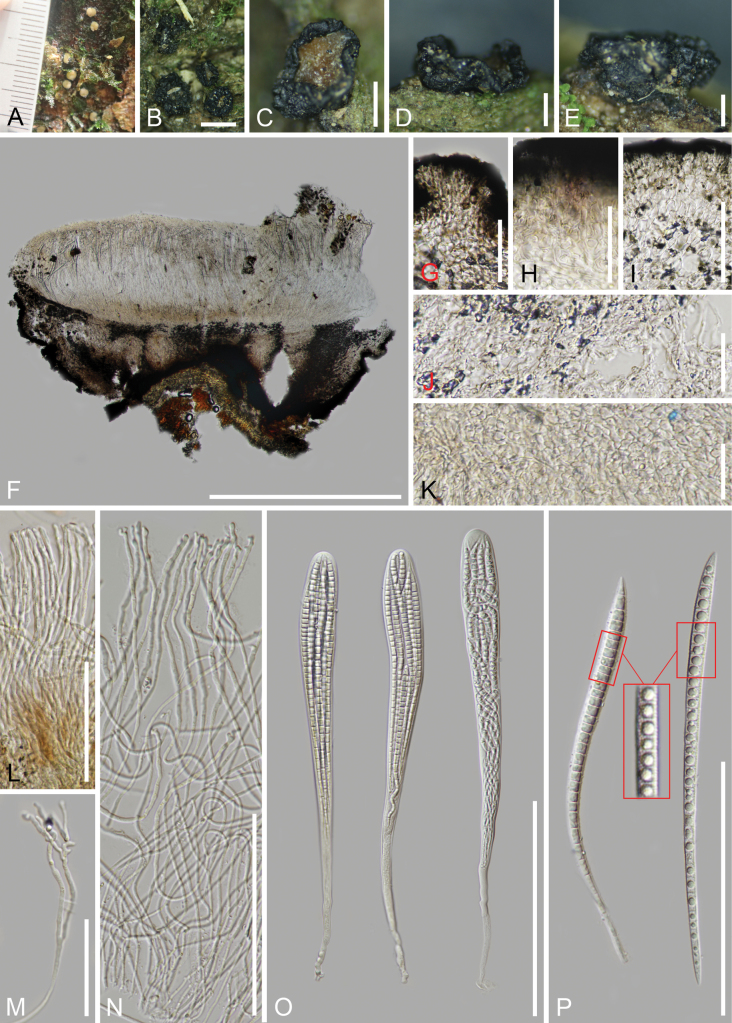
*Triblidium longisporum* (HKAS 145642, holotype). A. Fresh apothecia on the substrate; B–E. Dried apothecia on the substrate; F. Vertical section of an apothecium in 2% KOH reagent; G–I. Ectal excipulum in 2% KOH reagent; J. Medullary excipulum in 2% KOH reagent; K. Subhymenium; L. Short marginal paraphyses; M, N. Paraphyses in 2% KOH reagent; O. Asci in 2% KOH reagent; P. Ascospores (the left in 2% KOH reagent). Scale bars: 1 mm (B); 700 μm (C, F); 400 μm (D–E); 40 μm (G, H); 50 μm (I); 30 μm (J, K); 70 μm (L, M); 100 μm (N, P); 150 μm (O).

##### Holotype.

HKAS 145642.

##### Diagnosis.

It is similar to *T. stipitatum*, but *T. longisporum* has soft orange hymenium without stipes, simple basal stroma structures and slightly shorter asci and ascospores.

##### Description.

Saprobic on the bark of the living Fagaceae tree. **Sexual morph**: ***Apothecia*** 1.3–2.3(–3,1) mm wide (*x̄* = 1.8 μm, n = 20) when fresh, 1.1–1.9(–2.7) mm wide (*x̄* = 1.6 μm, n = 20), 0.5–0.8 μm high (*x̄* = 0.6 μm, n = 10) when dried, scattered, superficial, discoid, sessile, erumpent from the bark, initially growing as a cleistohymenial development, the hymenium tightly protected by excipulum when immature, splitting to expose hymenium by usually 6–8 teeth-like lobes in the surface in a humid environment, black (#4a4750) surface with polygonal areolae, becoming warty bulges after opening. ***Discs*** flat, circular to irregular shape, very soft orange (#dac696) when fresh, sub-circular to irregular shape, the edges irregularly curl towards the centre, translucent dark orange (#6d531f) when dried. ***Receptacles*** rough and black when fresh, sculptured with polygonal areolae or wrinkled on the surface when dried. ***Lips*** absent. ***Covering stroma*** 80–115(–135) μm thick, comprised of carbonised, textura angularis cells and the inner layers of hyaline, textura angularis to globulosa cells. ***Hymenium*** 330–400 μm (*x̄* = 360 μm, n = 10) thick, hyaline to pale yellow. ***Subhymenium*** 45–85 μm (*x̄* = 64 μm, n = 15) thick, comprised of hyaline, textura angularis cells, 4.0–9.2 μm (*x̄* = 6.1 μm, n = 40) in diam. ***Internal matrix of stroma*** (110–)140–270 μm (*x̄* = 195 μm, n = 30) thick, well-developed, comprised of hyaline, textura intricata hyphae, 1.6–3.3 μm (*x̄* = 2.2 μm, n = 40) in diam., non-gelatinous. ***Basal stroma*** (40–)56–128(–154) μm (*x̄* = 89 μm, n = 40) thick, well-developed, the outer layers comprised of carbonised, gelatinous, black red textura angularis cells, 3.1–6.0(–7.4) μm (*x̄* = 4.7 μm, n = 40) in diam., the inner layers comprised of hyaline cells, (3.8–)5.1–11.3(–15.7) μm (*x̄* = 7.2 μm, n = 70) in diam., partial elements orientated at a high angle to receptacle surface, non-gelatinous. ***Paraphyses*** 340–390 × 1.8–2.9 μm (*x̄* = 360 × 2.3 μm, n = 40) wide, hyaline with some tiny yellow oil drops, filiform, occasionally branched at the tips, aseptate, apically irregular-shaped and surrounded by a thin, gelatinous sheath. ***Asci*** ripening sequentially, 250–336 × 22–30 μm (*x̄* = 280 × 26 μm, n = 30), unitunicate, 8-spored, clavate, apically rounded without amyloid reaction in Melzer’s reagent, tapering to a fragile pleurorhynchous subtruncate base, croziers absent. ***Ascospores*** 160–196 × 5.9–10.5 μm (*x̄* = 176 × 7.9 μm, n = 40, Q = (16.1)18.8–30.3, Qm = 22.7 ± 3.4), overlapping fascicles, long acicular, transverse-septate, hyaline, 28–31-septate when mature, with a single oil drop in each cell, sharp ends, wide at the top and tapering downwards, thin and rough-walled with fine verrucae. **Asexual morph**: Undetermined.

##### Material examined.

China • Yunnan Province, Dali City, Jinguangsi Protection Zone, altitude 2,380 m, on the living bark of Fagaceae tree, 27 July 2024, Cuijinyi Li LCJY-1700 (HKAS 145642, holotype); • ibid., Cuijinyi Li LCJY-1691 (HKAS 145640, paratype).

##### Notes.

Our collection was placed sister to *T. stipitatum*, with the Maximum Likelihood bootstrap support of 86.5% in the SH-aLRT test, 92% in the UFB method and a Bayesian posterior probability of 1.0 (Fig. 1). *Triblidium longisporum* can be distinguished from other known species by its exceptionally long ascospores, with the exception of *T. stipitatum*. This species differs from *T. stipitatum* by its soft orange hymenium, lack of stipes, simple structures at the covering and basal stroma, smaller asci (250–336 μm vs. 272–355 μm) and shorter ascospores (160–196 μm vs. 187–226 μm). Based on the molecular analyses, the ITS sequence of *T. longisporum* exhibited a 11.4% difference with 34 gaps (60/523) to *T. stipitatum* (isolate: HKAS 145641), while the LSU sequence showed a 5.7% difference with one gap (48/838) to *T. stipitatum* (isolate: HKAS 145641) and the mtSSU sequence displayed a 3.7% difference with 10 gaps (31/831) to *T. stipitatum* (isolate: HKAS 145641).

#### ﻿ Triblidium stipitatum

Taxon classificationFungiRhytismatalesTriblidiaceae

C.J.Y. Li, K.W.T. Chethana & Q. Zhao sp. nov.

62B23841-9D42-5B7D-AC9A-3812D63AB7AA

Index Fungorum: IF904196

Facesoffungi Number: FoF18052

Fig. 4

##### Etymology.

The specific epithet refers to the presence of stipes, ‘*stipitatum*’ (lat.) = stipitate.

**Figure 4.**
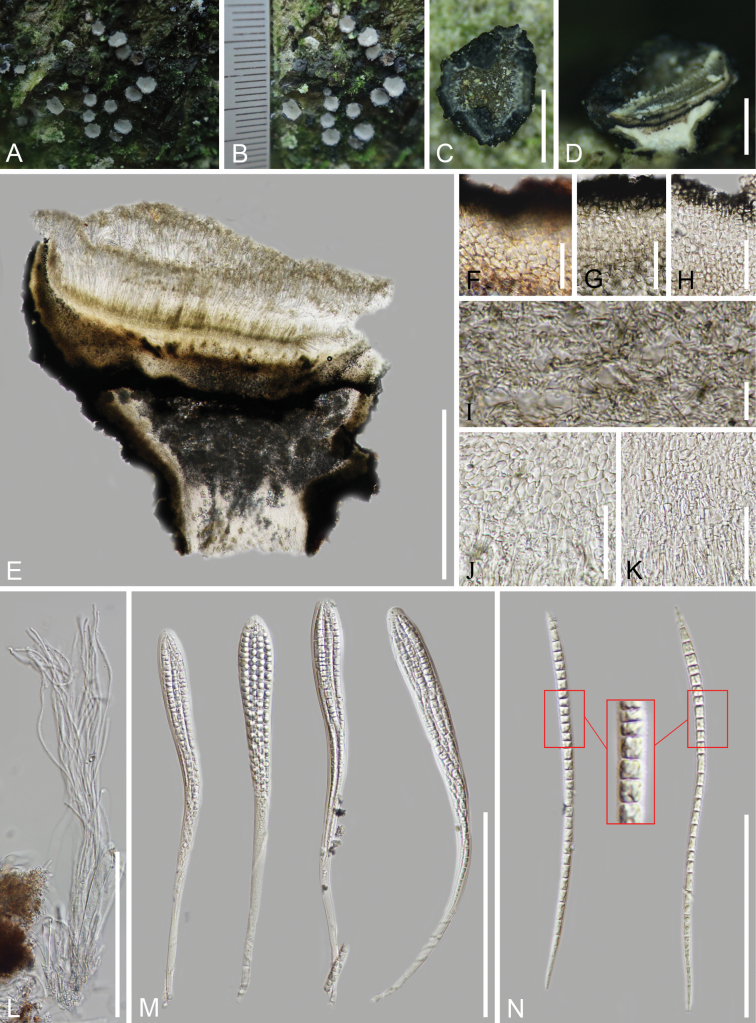
*Triblidium stipitatum* (HKAS 145641, holotype). A, B. Fresh apothecia on the substrate; C, D. Dried apothecia on the substrate; E. Vertical section of an apothecium in 2% KOH reagent; F–H. Ectal excipulum; G–H. Ectal excipulum in 2% KOH reagent; I. Medullary excipulum in 2% KOH reagent; J, K. Subhymenium in 2% KOH reagent; L. Paraphyses; M. Asci (the second in the water); N. Ascospores in 2% KOH reagent. Scale bars: 1 mm (C); 700 μm (D); 1 mm (E); 30 μm (F–H); 15 μm (I); 40 μm (J); 50 μm (K); 150 μm (L, M); 100 μm (N).

##### Holotype.

HKAS 145641.

##### Diagnosis.

It is similar to *T. longisporum*, but differs by having a stipitate, greyish-white disc, a basal stroma, consisting of three parts and slightly longer asci and ascospores.

##### Description.

Saprobic on the bark of the living Fagaceae tree. **Sexual morph**: ***Apothecia*** 1.9–3.2 mm wide (*x̄* = 2.6 μm, n = 30) when fresh, 1.6–2.6(–3.1) mm wide (*x̄* = 2.1 μm, n = 20), (0.5–)0.7–1.0 μm high (*x̄* = 1.6 μm, n = 20) when dried, scattered, superficial, cupulate, stipitate, erumpent from the bark, initially growing as a cleistohymenial development, obconical without a point, the hymenium tightly protected by excipulum when immature, splitting to expose hymenium by eight teeth-like lobes in the surface in a humid environment, black (#4a4750) surface with polygonal areolae, becoming warty bulges after opening. ***Discs*** flat to slightly detained in the centre, circular to irregular-shaped, greyish-white (#abb3b6) when fresh, triangular to angular-shaped, the edges curling towards the centre, desaturated dark green (#9eb07c) when dried. ***Receptacles*** rough and black when fresh, sculptured with polygonal areolae when dried. ***Stipes*** 0.5–1.0 mm wide, 0.6–1.0 mm high when dried, concolorous to the receptacles. ***Lips*** absent. ***Covering stroma*** 63–138 μm thick, comprised of carbonised, textura angularis cells and the inner layers hyaline, textura angularis to globulosa cells. ***Hymenium*** 378–432 μm (*x̄* = 399 μm, n = 20) thick, hyaline. ***Subhymenium*** 52–111 μm (*x̄* = 85 μm, n = 40) thick, comprised of hyaline, textura globulosa to angularis cells, 4.5–9.5(–11.3) μm (*x̄* = 7.1 μm, n = 80) in diam. ***Internal matrix of stroma*** 135–220 μm (*x̄* = 180 μm, n = 40) thick, well-developed, non-gelatinous, divided into three parts, part I near subhymenium comprised of dense and pale brown, textura intricata hyphae, 1.5–2.5 μm (*x̄* = 2.0 μm, n = 40) in diam.; part II in the middle, 37–74 μm wide, comprised of carbonised, black red (#28171a), textura angularis cells same as the ectal excipulum; part III (stipe) comprised of hyaline and densely parallel hyphae, mixed with large refraction resin materials, hyphae 1.7–2.5 μm (*x̄* = 2.1 μm, n = 60) in diam. ***Basal stroma*** 65–140 μm (*x̄* = 101 μm, n = 60) thick, well-developed, the outer layers comprised of carbonised, black red, textura angularis cells, 2.9–5.8 μm (*x̄* = 4.3 μm, n = 100) in diam., the inner layers comprised of hyaline cells, 5.5–10.3(–12.7) μm (*x̄* = 8.0 μm, n = 80) in diam., partial elements orientated at a high angle or vertical to receptacle surface, slightly gelatinous. ***Paraphyses*** 315–345 × 1.7–2.7 μm (*x̄* = 332 × 2.1 μm, n = 40) wide, hyaline, filiform, unbranched, aseptate, apically rounded, slightly swollen and waved, surrounded by a thin, gelatinous sheath. ***Asci*** ripening sequentially, 272–355(–373) × 21–30 μm (*x̄* = 312 × 25 μm, n = 40), unitunicate, 8-spored, clavate, apically ellipse, without amyloid reaction in Melzer’s reagent, tapering to a fragile pleurorhynchous, subtruncated base, croziers absent. ***Ascospores*** 187–226(–241) × 6.8–10.5 μm (*x̄* = 206 × 7.9 μm, n = 40, Q = (17.8)22.9–31.3, Qm = 26.3 ± 3.1), overlapping fascicles, long acicular, transverse-septate, hyaline, 24–33(–38)-septate when mature with a single oil drop in each cell, sharp ends, wide at the top and tapering downwards, thin and rough-walled with fine verrucae. ***Asexual morph***: Undetermined.

##### Material examined.

China • Yunnan Province, Dali City, Jinguangsi Protection Zone, altitude 2,380 m, on the living bark of Fagaceae tree, 27 July 2024, Cuijinyi Li LCJY-1695 (***holotype***HKAS 145641); • ibid., 24 July 2024, Cuijinyi Li LCJY-1642 (paratype HKAS 145639).

##### Notes.

*Triblidium stipitatum* is distinguished from all other known species by its well-developed stipe and the special excipulum structure. It is closely related to *T. longisporum*, based on both morphological and phylogenetic analysis, but can still be easily distinguished. *Triblidium stipitatum* can be differentiated from the latter species by the presence of stipes, greyish-white hymenium, a basal stroma consisting of three parts (from top to bottom: pale brown intricate hyphae, black red angularis cells and hyaline parallel hyphae mixed with large refraction resin materials), as well as slightly longer asci (272–355 μm vs. 250–336 μm) and ascospores (187–226 μm vs. 160–196 μm). The molecular analyses were shown in the note of *T. longisporum*.

## ﻿Discussion

Previous studies emphasised the use of variations in ascospore morphology for differentiating species within *Triblidium* (Eriksson 1992; Karakehian et al. 2019); however, this approach has often led to overestimations of taxonomic diversity and frequent revisions in classification (Lantz et al. 2011; Karakehian et al. 2019). The reliance on ascospore characteristics alone has proven insufficient for stable and accurate taxonomic delineation, highlighting the need for a more comprehensive evaluation of additional morphological and molecular features. Based on the current phylogenetic analysis, the elongated and slightly curved fusiform ascospores of *H. verrucosa* (now recognised as *T. verrucosa*) are distinct from the ellipsoid and muriform ascospores observed in other species, including the type species (Karakehian et al. 2019). Nevertheless, *T. verrucosa* is regarded as a member of *Triblidium* to maintain the monophyletic status of the genus. After incorporating two newly-described species with long acicular ascospores into the phylogenetic analysis, *T. verrucosa* continues to cluster within *Triblidium*. Similarly, *T. stipitatum*, *T. longisporum* and *T. caliciiforme* (the type species) cluster in the basal or lower clades. Despite its divergent morphology, *T. verrucosa* was still retained within *Triblidium* to maintain the monophyletic integrity of the genus (Lv et al. 2019).

We propose potential directions for future taxonomic studies here. Guo et al. (2024) suggested that *Triblidium* is a potential plant-associated ascomycete, with seven species reported from *Rhododendron* sp. (Ericaceae) and others isolated from branches of Fagaceae, Pinaceae and Salicaceae. As shown in Fig. 1, the phylogenetic tree reveals the following groups: Group I includes one species from Fagaceae, characterised by orange hymenium and ellipsoid, muriform ascospores; Group II comprises six species from *Rhododendron* sp., also with orange hymenium and ellipsoid, muriform ascospores; Group III contains a single species from Pinaceae, distinguished by orange hymenium, elongated and slightly curved ascospores with transverse septa, fine verrucae on the surface and distinctive round appendages at both ends; Group IV encompasses two species from Fagaceae, with greyish-white or pale orange hymenium and long acicular ascospores that exhibit transverse septa and fine verrucae; Group V includes one species from both Fagaceae and Salicaceae, notable for black hymenium and ellipsoid, muriform ascospores; Group VI represents an unidentified species from Fagaceae, exhibiting translucent yellow hymenium and ellipsoid, transversely stipitate ascospores. Although the ascospores in Groups III and IV are morphologically unique within *Triblidium*, they are phylogenetically closely related to species with ellipsoid ascospores in our study. Other micro-morphological characteristics did not facilitate delineating the clades. Thus, integrating plant associations, ascospore morphology and phylogenetic analysis supports these clades. The limited number of available species constrain comprehensive phylogenetic analyses. This study relied solely on ITS, LSU and mtSSU regions and the current database lacks information from additional genomic regions, such as protein-coding genes (e.g. *rpb*2, *tef*1), which are essential for comprehensive phylogenetic analyses and might lead to the further confirmation of the monophyly of *Triblidium* or potential division. Future research incorporating more diverse morphological and molecular data will likely yield more understanding of the interspecific relationships within *Triblidium*.

At the same time, it remains debatable whether excessive emphasis has been placed on the study of spore morphology. From a broader view, all members of the family share carbonised black ascomatal walls, which serve to protect the delicate spore layer in arid environments (Karakehian et al. 2019). However, variations in spore morphology may reflect diverse survival strategies. For example, muriform ascospores may exhibit greater adaptability in harsh terrestrial ecosystems; the large spores tend to be deposited over short distances and are less sensitive to desiccation and UV radiation, while small spores are more suited for long-distance dispersal via air or water. Thick-walled spores offer resistance to dry environments and the surface ornamentation can reduce settling velocities and influence trophic method (Karakehian et al. 2019; Quijada et al. 2022). Spore morphology could offer insights, potentially prompting a re-evaluation of current taxonomic classifications. This may result in the re-organisation of existing taxa and the establishment of additional genera within Triblidiaceae.

## Supplementary Material

XML Treatment for Triblidium daliense

XML Treatment for Triblidium longisporum

XML Treatment for Triblidium stipitatum

## ﻿Additional information

### Use of AI

No use of AI was reported.

### Author ORCIDs

Cui-Jin-Yi Li https://orcid.org/0000-0002-2805-7071

Qi Zhao https://orcid.org/0000-0001-8169-0573

Prapassorn Damrongkool Eungwanichayapant https://orcid.org/0000-0001-8005-4137

Feng-Ming Yu https://orcid.org/0000-0001-9133-8645

Kevin David Hyde https://orcid.org/0000-0002-2191-0762

Kandawatte Wedaralalage Thilini Chethana https://orcid.org/0000-0002-5816-9269

Wei-Wei Liu https://orcid.org/0000-0001-5670-606X

Dong-Mei Liu https://orcid.org/0000-0001-9190-8871

### Data availability

All of the data that support the findings of this study are available in the main text.
